# Scurvy and food selectivity in childhood: a case report

**DOI:** 10.31744/einstein_journal/2023RC0356

**Published:** 2023-08-28

**Authors:** Nicole Carvalho Xavier Micheloni da Silva, Paula Fraiman Blatyta Caselli, Chahine Pereira Marinho, Lucia Daihana Godoy Lopez, Fernanda Menezes Vasconcelos, Mariana Vicentin Nauff, Kamila Caixeta Gonçalves

**Affiliations:** 1 Hospital Municipal Dr. Moysés Deutsch Hospital Israelita Albert Einstein São Paulo SP Brazil Hospital Municipal Dr. Moysés Deutsch; Hospital Israelita Albert Einstein, São Paulo, SP, Brazil.

**Keywords:** Scurvy, Food preferences, Ascorbic acid, Feeding and eating disorders, Child, Adverse childhood experiences

## Abstract

Despite its rarity, symptomatic micronutrient deficiency remains a public health problem. Scurvy is the differential diagnosis for bleeding disorders and hematological and rheumatological diseases, especially in patients with eating disorders. However, it is unrelated to autism spectrum disorders or other neurodevelopmental disorders. A previously healthy 10-year-old boy living in São Paulo, Brazil, had a history of significant food selectivity unrelated to autism spectrum disorder, resulting in symptomatic ascorbic acid deficiency (scurvy). This resulted in pain and purpuric lesions on the lower limbs, gingival edema, bleeding during tooth brushing, asthenia, weakness, malaise, and sadness. Therefore, dietary anamnesis is important for routine monitoring of child growth and development. This process helps prevent nutritional deficiencies, facilitates early diagnosis of eating disorders, and enables multidisciplinary follow-up for these patients.

## INTRODUCTION

Scurvy is a nutritional disorder caused by an ascorbic acid deficiency. It was historically notorious among sailors but is a rare occurrence today. High-risk groups for scurvy in the modern world include people prone to malnutrition, psychiatric illnesses, eating disorders, limited food access, and malabsorptive states.^(1)^

The presenting patient had extreme dietary selectivity and presented with an eating disorder characterized by components of both anorexia nervosa and Avoidant Restrictive Food Intake Disorder (ARFID). These conditions were influenced by his socioeconomic status, which contributed to vitamin deficiency and the development of scurvy.

## CASE REPORT

**Figure 1:**
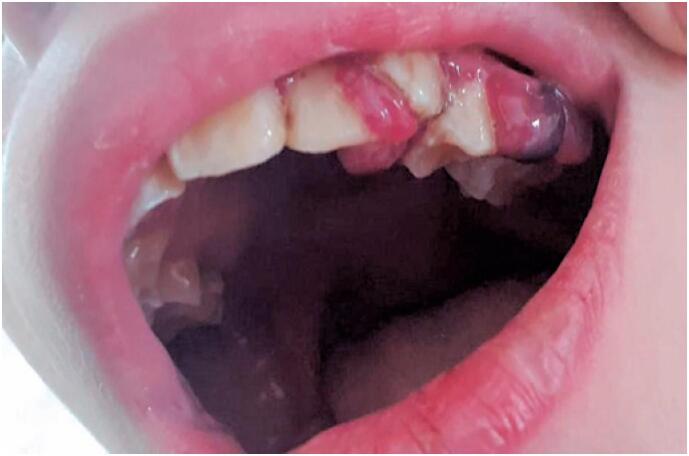
Swollen and inflamed gums

**Figure 2:**
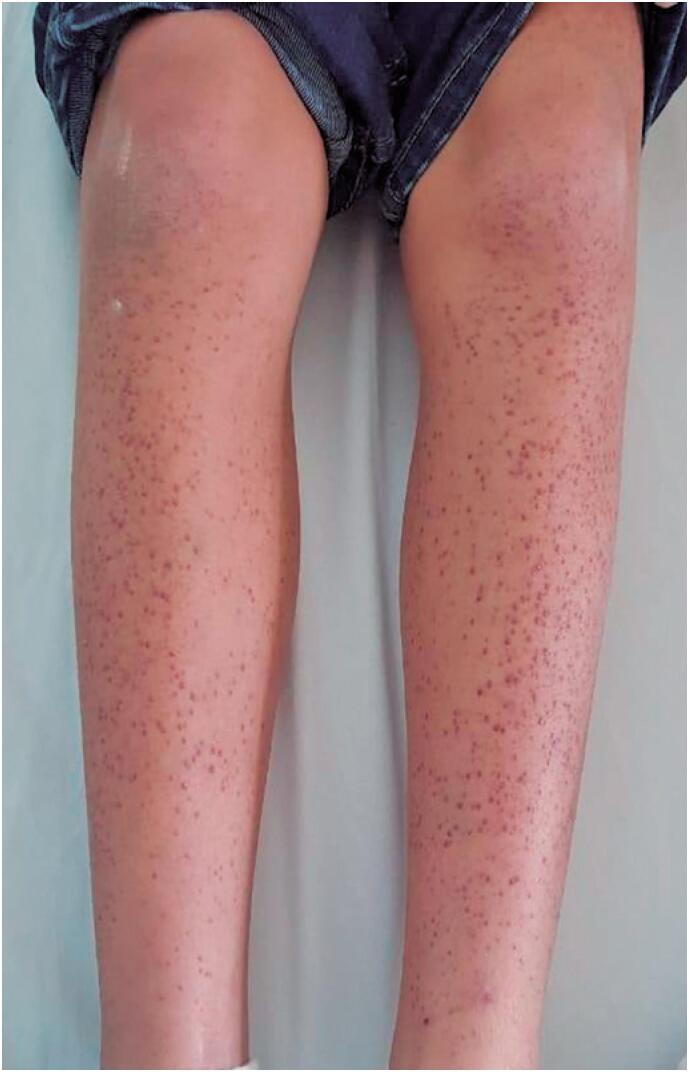
Purpuric lesions on lower limbs

A previously healthy 10-year-old boy was admitted to the emergency department due to a one-week history of pain and purpuric lesions on the lower limbs and symptoms of gingival edema and bleeding during tooth brushing. In addition, he complained of asthenia, weakness, malaise, and sadness. The child lived with his mother and three adult brothers in São Paulo, Brazil. The patient was in good general condition at admission, conscious, pale, eupneic, and afebrile. He had swollen and inflamed gums (Figure 1) and symmetrically distributed purpuric and palpable lesions on the lower limbs (Figure 2) without edema. Cardiovascular, respiratory, and gastrointestinal systems were normal. Anthropometry: weight 27.7kg and height, 138cm; body mass index, 14.57; fifth percentile, eutrophic according to the World Health Organization chart.

The first hypothesis during hospitalization for clinical evaluation was IgA vasculitis (Henoch-Schönlein purpura). An eating disorder was evident due to a current dietary intake of only rice, beans, and cookies. Approximately three years before hospital admission, the patient began exhibiting food selectivity that progressively worsened in 2020, with a reported weight loss of 25kg between March and June 2021.

The pediatric, nutrition, and psychiatry teams evaluated the patient. Considering the recent weight loss, food selectivity, and concern with weight gain and body appearance, the teams identified the risk of nutritional deficiencies, ARFID, and anorexia nervosa. Based on the presented clinical symptoms and the patient's dietary history, the initial treatment approach focused on addressing the patient's ascorbic acid deficiency (scurvy).

Laboratory tests revealed normal values for blood count, coagulation, renal function, electrolytes, liver function, bilirubin, total protein, albumin, iron, transferrin, folic acid, blood glucose, glycated hemoglobin, vitamin D, vitamin B12, total cholesterol and fractions, triglycerides, and thyroid hormones. Ascorbic acid levels were not measured at our hospital.

Coagulopathy and other nutritional deficiencies were excluded before initiating treatment for scurvy with ascorbic acid (100mg PO TID for one week, followed by 100mg PO once daily) until the symptoms improved. Patient referrals included nutrition services, children's psychiatric centers, and community health centers.

Seven days after hospital discharge and regular use of ascorbic acid, the patient showed improved diet acceptance, better mood, and the absence of asthenia, lower limb pain, and gingival lesions. There was also a significant regression of purpuric lesions on the lower limbs, confirming the diagnosis of ascorbic acid deficiency.

This case study was approved by the Ethics Committee of *Secretaria Municipal da Saúde* (CAAE: 60817722.5.0000.0086, # 5.677.587).

## DISCUSSION

Despite its rarity, symptomatic micronutrient deficiency remains a major public health concern. Even though micronutrients are required in small amounts, their deficiency can have detrimental effects on health if not managed effectively.^(2)^

Iron, iodine, folate, vitamin A, and zinc were the most common micronutrient deficiencies.^(2)^ Deficiency of ascorbic acid-a water-soluble vitamin-causes scurvy, characterized by gingivitis, abnormal wound healing, arthralgia, asthenia, and skin manifestations such as purpura.

Although less frequent in pediatric patients, researchers have reported ascorbic acid deficiencies in children with more restrictive diets. These patients often include children with intellectual disability, autism spectrum disorder (ASD), major eating disorders, or those living in poverty; also affected are children with iron overload due to medical conditions, such as sickle cell anemia.^(3)^

Childhood eating disorders can be triggered by various psychiatric or eating disorders such as ASD, anorexia nervosa, and ARFID, among others. These diagnoses are based on the American Psychiatric Association's Diagnostic and Statistical Manual of Mental Disorders, Fifth Edition (DSM-5), which categorizes eating disorders based on observed symptoms.^(4,5)^ The patient in our case study similarly exhibited extreme dietary selectivity and presented with a combination of anorexia nervosa and ARFID components due to his socioeconomic status. These factors contributed to vitamin deficiency and the development of scurvy.

Studies suggest that the pathogenesis of anorexia nervosa can include genetic and environmental factors, as well as changes in brain structure and function.^(6,7)^ Diagnostic criteria for anorexia nervosa include significant fear of gaining weight or becoming fat, or persistent behaviors that prevent weight gain despite being underweight; restriction of energy intake that leads to low body weight relative to the patient's age, sex, developmental trajectory, and physical health; distorted perception of body weight and shape; the undue influence of weight and shape on self-esteem; or denial of the medical severity of low body weight.^(7)^

Avoidant Restrictive Food Intake Disorder is an eating disorder characterized by a persistent failure to meet nutritional or energy needs, which can manifest as clinically significant weight loss or, in children, poor growth or failure to achieve the expected weight gain. Individuals with ARFID may depend on enteral feeding or nutritional supplements, experience significant nutritional deficiencies, and face marked interference with psychosocial functioning.^(7)^ While early onset anorexia nervosa and ARFID share common food restrictions, the underlying reasons for restrictions differ. In ARFID, the restriction may be influenced by factors such as the sensory characteristics of food, fear of the consequences of ingestion, or lack of interest in eating, whereas in early onset anorexia nervosa, the restriction stems from the fear of gaining weight.^(8)^

Ascorbic acid participates in fatty acid transport, collagen synthesis, neurotransmitter synthesis, prostaglandin metabolism, and nitric oxide synthesis.^(9)^ The human body does not produce ascorbic acid; therefore, it is an essential dietary micronutrient. The preliminary stages of ascorbic acid deficiency generate nonspecific symptoms, such as irritability, myalgia, arthralgia, weight loss, weakness, malaise, and lethargy. More advanced stages result in connective tissue defects, which can weaken the capillaries with subsequent hemorrhaging, bone damage, and impaired growth in children.^(10)^Cutaneous manifestations include follicular hyperkeratosis and perifollicular hemorrhage. Initially, petechiae appear flat and sparse but become confluent, leading to palpable purpura. The purpuric lesions observed in the patient suggest systemic vasculitis, with IgA vasculitis as the primary differential diagnosis. The oral manifestations included swelling, purplish and friable gums, spontaneous bleeding after minor trauma, eventual tooth loss, and secondary bacterial infection.^(10)^

Edema, weakness, and fracture of long bones may occur in addition to intramuscular and subperiosteal hemorrhages. The most common radiographic findings are osteopenia and sclerosis. In infants, musculoskeletal impairments begin with irritability, pain during movement, and delayed growth.^(9,10)^

Scruvy manifests when the body's pool of ascorbic acid (typically approximately 1,500mg) falls below 300mg.^(1)^ This measurement was not available at our hospital; therefore, the scurvy diagnosis, in this case, was based on the clinical presentation, dietary history, and rapid remission of manifestations after diagnostic and therapeutic intervention with ascorbic acid replacement.

One limitation of our study is the absence of a laboratory methodology for ascorbic acid dosing, which prevented us from confirming the micronutrient deficiency. However, we collected various other laboratory test results that were within the normal range; our patient improved with our nutritional recommendations, which supported our diagnosis of ascorbic acid deficiency.

## CONCLUSION

Despite the low prevalence of ascorbic acid deficiency, it remains a differential diagnosis for bleeding disorders and hematologic and rheumatologic diseases, particularly in patients with associated eating disorders. However, it is unrelated to autism spectrum disorder or other neurodevelopmental disorders.

In this case, diagnostic suspicion arose based on the clinical presentation and the patient's history of food selectivity, which was confirmed after diagnostic and therapeutic tests. Dietary anamnesis is important for the routine monitoring of child growth and development. It helps in the prevention of nutritional deficiencies, early diagnosis of eating disorders, and ensures comprehensive multidisciplinary follow-up for these patients.
